# A Case Report of Delayed Post-operative Angioedema Associated With Angiotensin-Converting Enzyme Inhibitor Use

**DOI:** 10.7759/cureus.18800

**Published:** 2021-10-15

**Authors:** Robert J Dabek, Nancy A Pina, Benjamin A Sheber, Anna Axentiev, Michael C Scott

**Affiliations:** 1 General Surgery, Ascension St. Agnes Hospital, Baltimore, USA; 2 General Surgery, Ross University School of Medicine, Bridgetown, BRB; 3 Critical Care, Ascension St. Agnes Hospital, Baltimore, USA

**Keywords:** facial angioedema, angioedema, post-operative complication, drug-induced angioedema, angiotensin converting enzyme inhibitors

## Abstract

Angiotensin-converting enzyme inhibitors are known to precipitate angioedema. Drug-induced angioedema is rare in the perioperative setting. Even fewer cases described hours following a minor procedure. In this case report, we present a 45-year-old female who developed drug-induced angioedema hours following an obstetric procedure.

## Introduction

Angioedema is characterized by asymmetrical non-pitting swelling of the subcutaneous tissues. It commonly affects the lips, tongue, face, upper airway, and gastrointestinal tract. In some cases, angioedema can be life-threatening [1]. Angioedema may be mast-cell mediated, bradykinin mediated, hereditary, or idiopathic. Angiotensin-converting enzyme (ACE) inhibitor exposure is the most common culprit in drug-induced angioedema, accounting for 20%-40% of emergency room visits due to angioedema each year [2-4]. ACE inhibitor-induced angioedema results from defective degradation of bradykinin, a vasoactive peptide. Angioedema is rare in the perioperative setting, with several cases reported during head and neck surgery or in the context of trauma [5]. In this case report, we describe a case of delayed drug-induced angioedema presenting after an elective obstetric procedure.

## Case presentation

A 45-year-old female presented to the outpatient surgical clinic with dysfunctional uterine bleeding. Her medical history was significant for hypertension, hyperlipidemia, and asthma. Home medications included lisinopril (40 mg daily), chlorthalidone (25 mg daily), atorvastatin (40 mg daily), aspirin (325 mg daily), and a single dose of misoprostol (200 mcg) prescribed preoperatively in anticipation of an elective obstetric procedure. The patient underwent hysteroscopy with dilation and curettage under general anesthesia. After induction, a grade 1 view was obtained and the patient was intubated using a 7.0 mm endotracheal tube without complications. The procedure was completed as planned and the patient was extubated and brought to the post-anesthesia care unit (PACU). She had an uneventful recovery in the PACU and was discharged two hours post-operatively. Four hours after discharge, she noted the onset of facial swelling, dyspnea, and hoarseness of voice. These symptoms worsened over the following six hours, prompting her to seek medical attention.

On arrival to the emergency department, she was hypertensive (186/104 mmHg), tachycardic (107 beats per minute), tachypneic (33 breaths per minute), with an oxygen saturation of 96%. On examination, she had edema of the upper lip and uvula; however, she remained afebrile and had no skin flushing, urticaria, pruritus, or gastrointestinal disturbances. She was diagnosed with angioedema. A decision to intubate the patient was made, and she was admitted to the intensive care unit (ICU) for further management, including sedation, dexamethasone, and diphenhydramine. Lisinopril was discontinued at this time. On post-operative day 3 (ICU day 2), she was found to have decreased facial edema, and she was noted to have a significant air leak after deflation of the endotracheal tube cuff. She was extubated uneventfully and remained under ICU care overnight. Speech and language pathology evaluated the patient and she was cleared to resume a regular diet. At this time, she was transferred to a general medicine unit and discharged home in good condition the following day.

## Discussion

ACE inhibitors are the most common culprit of drug-induced angioedema, a potentially life-threatening swelling of the subcutaneous tissues. Angioedema commonly involves the face, oropharynx, and gastrointestinal tract. An observational study assessing angioedema incidence demonstrated that 55% of cases occurred within 90 days of ACE inhibitor initiation; however, the risk of developing angioedema beyond 90 days remained 0.1% to 0.7% [6,7]. 

Angiotensin-converting enzyme (ACE) is responsible for the degradation of bradykinin, a vasoactive peptide. ACE inhibitors, therefore, cause an increase in the levels of serum bradykinin, a finding also described in ACE inhibitor-induced angioedema [8]. Elevated bradykinin induces vasodilation, increased vascular permeability, and protein extravasation into the extravascular space contributing to the development of angioedema [9]. Other forms of angioedema include mast-cell mediated, hereditary, or idiopathic. Initial signs include facial, lip, and uvula swelling as well as dyspnea, all seen in our patient. Although an allergic reaction and anaphylaxis were on the differential, the absence of urticaria and pruritis disfavored these diagnoses.

The risk factors for developing ACE inhibitor-induced angioedema include age greater than 65 years, African American or Hispanic race, female sex, concomitant use of aspirin or nonsteroidal anti-inflammatory drugs, previous episodes of angioedema, and seasonal allergies. In addition, risk factors for severe presentation include American Society of Anesthesiologists (ASA) class III-V, smoking history, cardiopulmonary disease, Hispanic race, and edema in the deeper tissues of the aerodigestive tract at first presentation [10]. Our patient had multiple risk factors for the development of severe angioedema, including female sex, aspirin use, Hispanic race, and ASA class III. Lisinopril, an ACE inhibitor, was determined to be the causative agent as opposed to misoprostol, which was newly prescribed preoperatively as misoprostol-induced angioedema is described at the risk of less than 0.01%, whereas the risk of ACE inhibitor-induced angioedema is described at 0.1% to 0.7%.

Immediate management of angioedema focuses on airway protection as respiratory failure may ensue secondary to airway edema and complete obstruction. Patients with stridor, accessory muscle use, excessive drooling of saliva, or severe tongue or mouth-floor edema should be immediately intubated. In patients, who present with hoarseness, odynophagia, or dyspnea, fiberoptic nasopharyngolaryngoscopy (NPL) can be a useful technique to evaluate for laryngeal edema [11]. Patients should then be admitted to the intensive care unit for close airway monitoring. Discontinuation of the offending agent is essential to prevent a recurrence. Although ACE inhibitor-induced angioedema is self-limiting, adjuvant treatment often includes the use of corticosteroids and antihistamines, as these drugs can treat allergic and idiopathic causes of angioedema if the diagnosis remains obscured [11,12].

## Conclusions

Although the risk of ACE inhibitor-induced angioedema is well described, cases in the perioperative period are rare. Further investigation into the incidence of perioperative angioedema is required in order to establish recommendations for prophylactic discontinuation of this class of medication prior to an operation. In the meantime, the severity of this presentation warrants clinician awareness across specialties. We recommend educating patients on the presenting symptoms in drug-induced angioedema. This knowledge is intended to prevent delayed clinical presentation, associated with an increased risk of mortality due to respiratory failure from airway obstruction. Additionally, patients with documented ACE inhibitor-induced angioedema must discontinue this class of medications as subsequent episodes are of rapid onset and increased severity.
